# Quantifying the contribution of transcription factor activity, mutations and microRNAs to CD274 expression in cancer patients

**DOI:** 10.1038/s41598-022-08356-0

**Published:** 2022-03-14

**Authors:** Imke B. Bruns, Joost B. Beltman

**Affiliations:** Division of Drug Discovery and Safety, Leiden Academic Centre for Drug Research, Leiden University, Leiden, The Netherlands

**Keywords:** Cancer, Computational biology and bioinformatics, Biomarkers, Oncology

## Abstract

Immune checkpoint inhibitors targeting the programmed cell death protein 1 (PD-1)/programmed cell death protein ligand 1 (PD-L1) axis have been remarkably successful in inducing tumor remissions in several human cancers, yet a substantial number of patients do not respond to treatment. Because this may be partially due to the mechanisms giving rise to high PD-L1 expression within a patient, it is highly relevant to fully understand these mechanisms. In this study, we conduct a bioinformatic analysis to quantify the relative importance of transcription factor (TF) activity, microRNAs (miRNAs) and mutations in determining PD-L1 (CD274) expression at mRNA level based on data from the Cancer Genome Atlas. To predict individual CD274 levels based on TF activity, we developed multiple linear regression models by taking the expression of target genes of the TFs known to directly target PD-L1 as independent variables. This analysis showed that IRF1, STAT1, NFKB and BRD4 are the most important regulators of CD274 expression, explaining its mRNA levels in 90–98% of the patients. Because the remaining patients had high CD274 levels independent of these TFs, we next investigated whether mutations associated with increased CD274 mRNA levels, and low levels of miRNAs associated with negative regulation of CD274 expression could cause high CD274 levels in these patients. We found that mutations or miRNAs offered an explanation for high CD274 levels in 81–100% of the underpredicted patients. Thus, CD274 expression is largely explained by TF activity, and the remaining unexplained cases can largely be explained by mutations or low miRNA abundance.

## Introduction

Our immune system is a complex network of cell types and molecular components that protect the human body from exogenous invaders such as viruses, bacteria, fungi, and toxins. Immune responses in healthy individuals are also tightly regulated to prevent conditions such as allergies or auto-immune diseases^1^. Such regulation is mediated in part by immune checkpoints, which act as gatekeepers of immune responses, and consist of co-stimulatory immune receptors that stimulate immune reactions when interacting with their ligand, and co-inhibitory immune receptors, which inhibit immune reactions^2,3^.

Numerous inhibitory immunoreceptors have been identified as playing a role in cancer, including, but not limited to CTLA-4, LAG3, and BTLA^4,5^. A highly important co-inhibitory immune checkpoint is programmed cell death protein (PD-1), which interacts with its main ligand, programmed cell death ligand 1 (PD-L1), also known by its gene name CD274^6^. PD-1 is highly expressed on the surface of activated CD8 + T cells, while PD-L1 is mainly expressed on antigen-presenting cells, such as macrophages and dendritic cells, and tumor cells^7^. When PD-L1 binds to its receptor PD-1, PD-1 forms clusters with T cell receptors, becomes phosphorylated, and binds to the Src homology (SH2) domains of SH2-containing phosphatase 2 (SHP2). SHP2 dephosphorylates the CD3 complex, which is responsible for the activation of both CD4^+^ and CD8^+^ T cells^8^. As a consequence, the interaction of PD-1 and its ligand PD-L1 results in the inhibition of T cell activation, proliferation, and secretion of cytotoxic granules, which thus limits anti-tumor responses^9,10^. This has resulted in an enormous interest in targeting the PD-1/PD-L1 pathway to develop new cancer treatments^11^.

**Figure 1:**
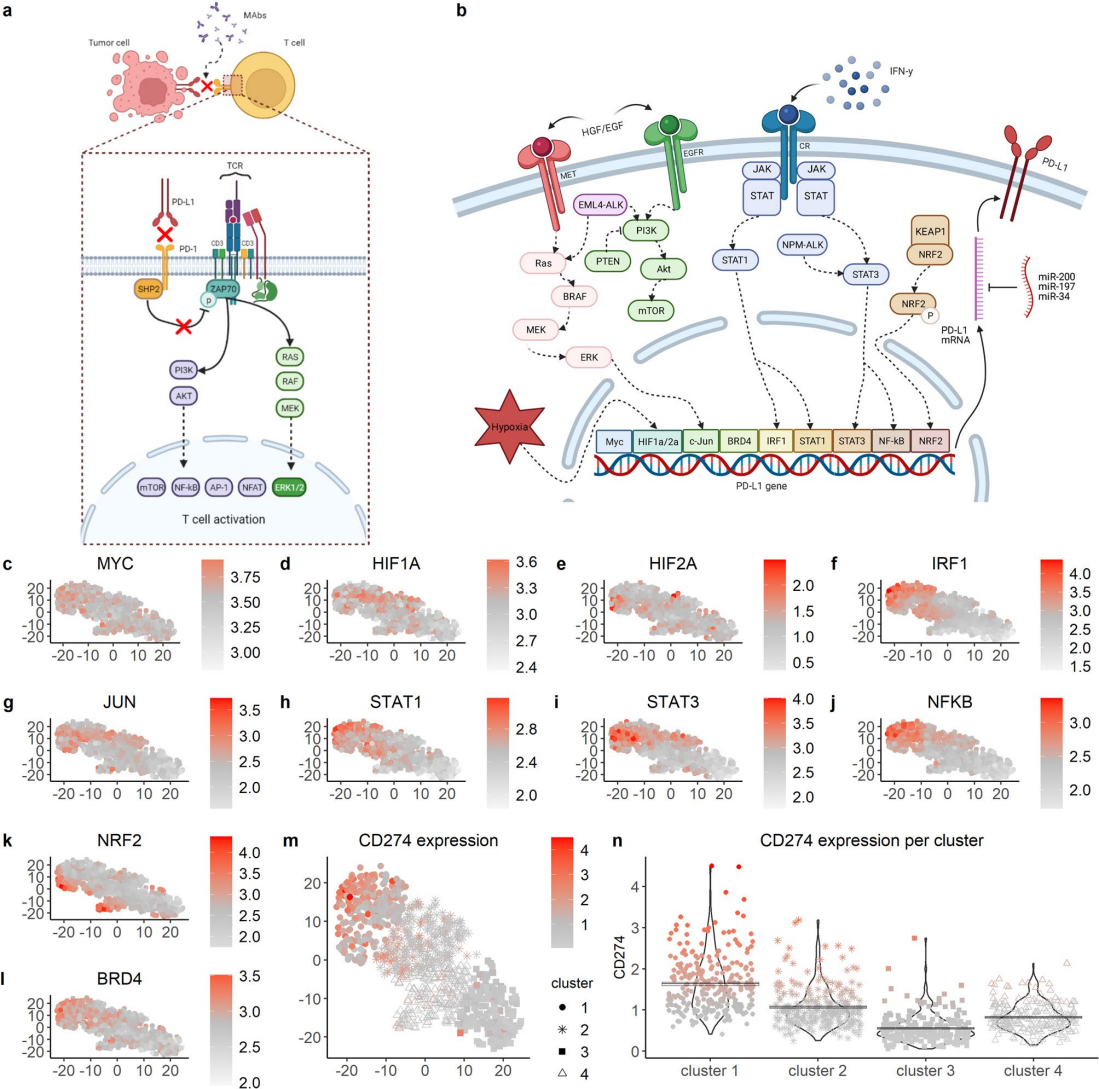
Relationship between transcription factor activity and CD274 expression. (**a**,**b**) Schematic representations of binding and consequences (red crosses) of PD-1/PD-L1 inhibition (**a**) and of transcriptional and post-transcriptional regulation of PD-L1 (**b**). Both schemes created with BioRender.com. (**c–l**) t-SNE plots based on TCGA data from 960 BRCA patients, taking the activity of the indicated transcription factor estimated by target gene expression as a basis to develop the t-SNE. Color indicates transcription factor activity. (**m**) CD274 expression in the t-SNE plot (note that the t-SNE was developed on the basis of TF activity and not on CD274 expression). (**n**) CD274 expression per k-means cluster (clusters based on the t-SNE plot in (**m**)).

Inhibiting the interaction between PD-1 and PD-L1 prevents SHP2 related dephosphorylation of the CD3 complex, resulting in an enhanced T cell function and anti-tumor activity^6,12,13^ (Fig. 1a). Monoclonal antibodies (MAbs) targeting PD-1 and PD-L1 have been remarkably successful in inducing remissions in various tumors. To date, 6 MAbs against human PD-1 or PD-L1 have been approved by the Food and Drug Administration (FDA) for cancer immunotherapy: *Pembrolizumab*, *Nivolumab*, and *Cemiplimab* targeting PD-1, and *Atezolizumab*, *Durvalumab*, and *Avelumab* targeting PD-L1. However, despite the clinical benefits, a significant number of patients do not respond or do not show long-lasting remission after treatment^14^. Individual environmental factors such as smoking or food intake and intratumoral heterogeneity create a unique therapeutic landscape, which causes differences in response between patients receiving PD-1/PD-L1 blockade therapy^15^. The overall response rate to PD-1/PD-L1 blockade differs between cancer types, and ranges from 13% in head and neck carcinoma to 45% in melanoma^16,17^.

Considering that immune checkpoint inhibitors (ICIs) are expensive and may cause immune-associated adverse events, it is of high importance to define biomarkers identifying a subset of patients suitable for PD-1/PD-L1 blockade therapy^18,19^. Despite some limitations, the assessment of PD-L1 levels via immunohistochemistry (IHC) staining in formalin-fixed paraffin-embedded tumor tissue samples is currently the only biomarker approved for clinical use, and is fully integrated into routine clinical practice^20,21^. To identify additional biomarkers for classification of responsive tumors, it is important to understand the complicated regulation of PD-L1 expression. This expression is controlled at mRNA and protein level, including the amount at the cell membrane^22^. First, the pathways JAK/STAT, PI3K/Akt/mTOR, ERK-MAPK and EML4-ALK, and the associated TFs Myc, HIF1A/2A, c-Jun, IRF1, STAT1, STAT3, NFKB, BRD4, and NRF2 lead to altered PD-L1 transcription^23–25^ (Fig. 1b). However, the relative importance of these TFs in determining PD-L1 expression is currently unknown. A second factor affecting PD-L1 expression is post-transcriptional regulation by microRNAs (miRNAs), which can bind to the 3’ untranslated region (3’UTR) of target mRNAs, thereby inducing mRNA degradation and translational repression^26^. Thus, low levels of specific miRNAs downregulation PD-L1 expression, such as miR-200, miR-197, and miR-34^27^, could explain elevated PD-L1 expression. Indeed, particular miRNAs were lower expressed in PD-1/PD-L1 blockade therapy responders compared to non-responders^28^. A third factor affecting PD-L1 expression involves genetic alterations within tumors. For example, epidermal growth factor receptor (EGFR) mutations are associated with upregulated PD-L1 expression^29,30^, and consequently the overall response rate (ORR) to the PD-L1 inhibitor *Atezolizumab* in EGFR-mutant patients is higher (31%) compared to the EGFR wild-type subgroup (22%)^31^. Similarly, patient subgroups with TP53 and KRAS mutations have an increased PD-L1 expression and a prolonged progression-free survival (PFS) after PD-1 inhibitor therapy^32^.

Here, we quantify the relative importance of CD274 regulation at mRNA level by TF activity, miRNA regulation, and mutations using a bioinformatic analysis based on data from The Cancer Genome Atlas (TCGA). First, we singled out the most important TFs regulating CD274 using multiple linear regression (MLR) models. This analysis indicated that IRF1, STAT1, NFKB, and BRD4 are the most important regulators of CD274 expression, leading to its correct prediction in ~ 90–98% of the patients. Second, we aimed to identify factors capable of explaining the high CD274 levels in patients with underpredicted CD274 expression based on the MLR models. We identified 36, 44, and 81 genetic alterations, and 29, 89, and 81 miRNAs that are associated with CD274 upregulation in breast invasive carcinoma (BRCA), lung adenocarcinoma (LUAD), and skin cutaneous melanoma (SKCM), respectively. Mutations in those critical genes, and low expression levels of those critical miRNAs could explain 81%, 83%, and 100% of the underpredicted patients.

## Results

### TF activity based on target gene expression corresponds with PD-L1 expression in breast cancer patients

Multiple TFs are known to contribute to regulation of PD-L1 expression^23–25^ (Fig. 1b), yet it is unclear what their relative importance is in determining PD-L1 expression in cancer patients, and how variable this is amongst human cancers. We therefore set out to quantify to what extent the PD-L1 mRNA level (CD274) in cancer patients can be predicted by activity of the relevant TFs MYC, HIF1A/2A, JUN, IRF1, STAT1/3, NFKB, and NRF2. To this purpose, we investigated mRNA expression in BRCA patients in TCGA data. Because the activity of many TFs depends on the nuclear presence of these proteins, their expression at mRNA level is not always representative for their activity. Therefore, we instead used sets of known target genes as proxies for TF activity, with target genes based on DoRothEA^33^ (note that for BRD4 no information was available in DoRothEA, hence we based BRD4 target genes on a recent review^34^)*.* We subsequently removed target genes that overlapped between TFs (including CD274 itself; for resulting genes see Supplementary file 2, Sheet1) to ensure that TF activity is estimated on the basis of target genes as specific as possible. As expected, the average expression of the selected target genes correlated reasonably well with the mRNA abundance for a subset of TFs. For example, for IRF1 this is expected because IRF1 transcription and translation both increase in response to IFN-γ signaling^35^ (Supplementary file 1, Fig. S1). For other TFs such as NRF2 the correlation was much less clear, likely because NRF2 activity depends on translocation of NRF2 protein to the nucleus rather than on its own transcript levels. In some cases (e.g., STAT3, NFKB)), the correlation between TF activity as estimated from target gene expression and CD274 expression improved substantially compared to using the mRNA expression of the TF directly (Supplementary file 1, Fig. S2), suggesting that our approach to estimate TF activity is valid. In most other cases the resulting correlations from the two approaches remained similar (e.g., IRF1).

Subsequently, we used an unsupervised t-SNE analysis to investigate potential patterns in the activity of TFs (as estimated by target gene expression) known to target PD-L1. Although there were no clear t-SNE clusters, some of the TFs, including IRF1, STAT1, and NFKB, exhibited an increased expression in the upper left corner (Fig. 1c–l). Interestingly, the expression of PD-L1 followed a similar pattern (Fig. 1m), while its expression data were not used in the generation of the t-SNE plots. Indeed, CD274 expression levels were substantially different between clusters created by K-means clustering on the basis of the two t-SNE variables, with the highest CD274 expression in cluster 1 and the lowest in cluster 4 (Fig. 1n). This analysis indicates that there is a clear relationship between the activity of some of the TFs and the expression of PD-L1.

### Multiple linear regression models identify cancer-specific CD274 regulation

To explore the relationships between TFs known to regulate PD-L1, and CD274 expression in a quantitative manner, we constructed multiple linear regression (MLR) models. We extended our analysis to include three human cancer types (BRCA, LUAD and SKCM) for which large patient numbers were available in TCGA. As before, we used TF activities estimated on the basis of average target gene expression as input for the MLR analysis. Using a bounded variable least squares (BVLS) solver, we selected only the TFs with a positive effect on PD-L1. We accepted negative coefficients solely for BRD4, since this TF may inhibit CD274 expression (note that reports are conflicting)^23–25^. TFs with non-zero values and a significant (*p* < 0.001) effect on PD-L1 expression were included in the final MLR model. As a side note, although we utilized only fully independent target genes in our estimates of TF activity, potential multicollinearity of the involved genes might still weaken the model’s generalization ability^36^. However, multicollinearity analysis by calculation of the Variation Inflation Factor (VIF) values of the model’s independent variables resulted in VIFs lower than 4, indicating that the selected descriptors were not highly correlated (Supplementary file 1, Table S1).

The final model was highly significant (*p* = 2.2e−16), and for all three cancer types IRF1 was identified as a significant explanatory variable for CD274 expression (Table 1, left 3 columns). Interestingly, the contribution of IRF1 to CD274 expression relative to that of other significant TFs differed amongst cancer types (15% [SD: 1.4] in BRCA, 26% [SD: 2.1] in LUAD, and 56% [SD: 3.9] in SKCM). Other significant explanatory variables included STAT1 in BRCA (relative contribution of 51% [SD: 1.9]) and LUAD (relative contribution of 60% [SD: 2.6]), and NFKB in BRCA (relative contribution of 34% [SD: 1.7]) and SKCM (relative contribution of 44% [SD: 3.9]). Only in LUAD patients, the analysis identified HIF1A (relative contribution of 52% [SD: 3.7]) as a variable with a positive effect, and BRD4 (negative contribution of -39% [SD: 3.7]) as a variable with a negative effect on CD274 expression. The finding that HIF1A is an important factor in CD274 expression for LUAD patients is consistent with earlier published findings that in both small cell and non-small cell lung cancers HIF1A levels are typically high^37^. This could in part be due to cigarette smoking which is linked to HIF1A activation^38,39^. However, within the TCGA LUAD cohort there was no significant difference in HIF1A expression (*p* = 0.077; Supplementary file S1, Fig. S3A), or in HIF1A activity as estimated by target gene expression (*p* = 0.812; Supplementary file S1, Fig. S3B) between smokers and non-smokers. Note that this absence of a significant difference could be due to the small size of the subsets of patients identified as smokers and non-smokers, which also precluded a meaningful separate MLR analysis on these subpopulations.

**Table 1:** Fitted coefficients and *p*-values of linear regression model per cancer type.

	BRCA		LUAD		SKCM		Generalized	
	Reg. coef	*p*-value	Reg. coef	*p*-value	Reg. coef	*p*-value	Reg. coef	*p*-value
(Intercept)	− 4.0473	2e−16	− 6.5966	2e−16	− 2.9561	2e−16	− 2.8544	2e−16
MYC	–	–	–	–	–	–	–	–
HIF1A	–	–	1.3564	2.18e−13	–	–	–	–
HIF2A	–	–	–	–	–	–	–	–
JUN	–	–	–	–	–	–	–	–
IRF1	0.2768	7.79e−8	0.7081	1.95e−8	0.8639	2e−16	0.6488	2e−16
STAT1	1.0254	2e−16	1.9765	2.12e−10		–	0.5501	3.95e−6
STAT3	–	–	–	–	–	–	–	–
NFKB	0.7122	2e−16	–	–	0.8186	2.66e−11	0.8478	2e−16
NRF2	–	–	–	–	–	–	–	–
BRD4	–	–	− 1.0808	2.69e−7	–	–	− 0.4198	4.85e−5
R^2^	0.5987	2.2e−16	0.5115	2.2e−16	0.5666	2.2e−16	0.5431	2.2e−16

A missing regression coefficient (i.e., a dash) indicates that the given transcription factor was not included in the model.

**Figure 2:**
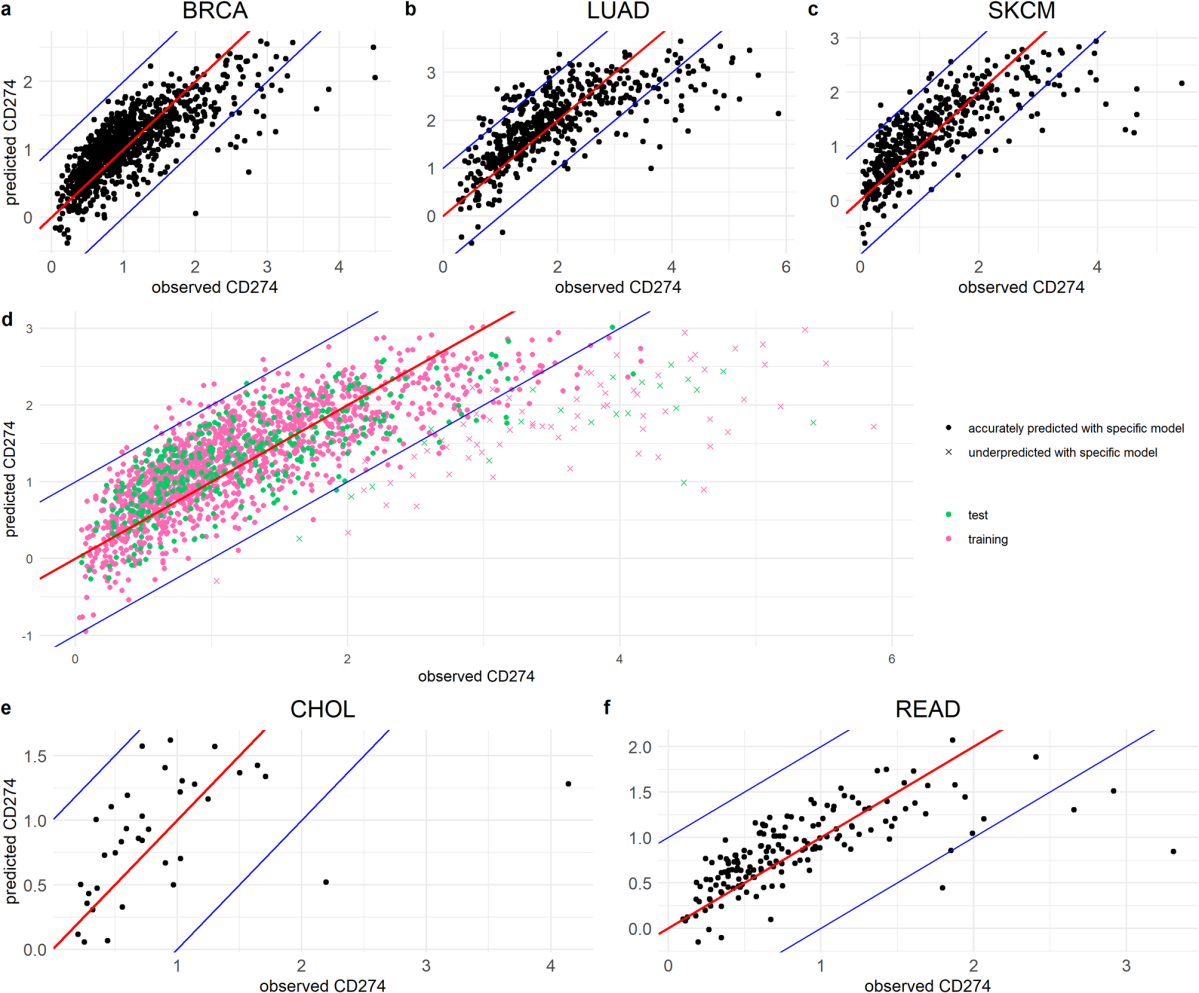
Multiple linear regression models correctly predict CD274 expression based on transcription factor activity in various human cancers. (**a–f**) Comparison of CD274 expression predicted by MLR (vertical axes) and observed CD274 expression (horizontal axes) for BRCA (**a**), LUAD (**b**) and SKCM (**c**) patients, and for a generalized MLR based on a training set of combined BRCA, LUAD and SKCM data applied to a test set of these combined data (**d**), or applied to CHOL (**e**) and READ (**f**) patients. In (**d**), differences compared to the specific models from (**a–c**) are indicated by symbol and color.

We used the final MLR models to predict CD274 expression for each individual patient and compared this to the actually observed CD274 expression in the 3 cancer types (Fig. 2a–c). In the large majority of patients, predictions were highly accurate, (observations within the blue lines implies that the prediction deviates by maximally two-fold), indicating that the identified small subset of TFs could quantitatively explain the observed CD274 expression. There was a small group of patients in whom CD274 expression was more than two-fold underpredicted, which we hereafter refer to as the ‘underpredicted group’. There was also a very small ‘overpredicted group’, yet in the following we solely focus on the underpredicted group. This underpredicted group consists of 2.2%, 9.6%, and 4.9% of all patients in BRCA, LUAD, and SKCM, respectively. In conclusion, we found IRF1 to be a general predictor for CD274 expression in all three studied cancer types, and STAT1, NFKB, HIF1A and BRD4 to be predictors for some of these cancer types. Based on this small set of TFs, CD274 expression could be explained in ~ 90–98% of patients, indicating that activity of these TFs is by far the most important determinant of CD274 expression.

### A generalized multiple linear regression model predicting CD274 expression extrapolates to other cancers

The MLR models developed so far were based on hundreds of patients per cancer type, which allowed us to identify differences in the importance of CD274-regulating TFs. For other cancer types typically much less patient data is available because these cancers are less common and possibly also studied less frequently. Therefore we asked whether a generalized MLR model based on the large number of patients amongst the three cancer types (in total 1907 patients) studied so far could be extrapolated to accurately predict PD-L1 expression in patients with other cancer types, thereby identifying patients with a high CD274 expression that cannot be explained by TF activity. We thus combined the BRCA, LUAD, and SKCM data, and randomly divided it into a training set of 1525 patients (80%), and a test set of 382 patients (20%). We repeated this procedure with different training sets of the same size to compare the statistics and coefficients of the final models. In all cases, the MLR models were of high significance (*p* = 2.2e−16) and presented exactly the same independent variables (IRF1, STAT1, NFKB, BRD4) as significant predictors for CD274 expression (Table 1, rightmost column). Overall, the prediction of CD274 expression gave similar results in cancer-specific models and the generalized model (Fig. 2d). Only 1.5% of the patients was predicted in the underpredicted group with the general model, while they were predicted in the accurately predicted group with the specific model, providing confidence in the potential for extrapolation to other human cancers.

To test whether the generalized MLR is able to predict CD274 levels in cancer types other than BRCA, LUAD, or SKCM, we used data from a total of 36 patients with cholangiocarcinoma (CHOL), and 165 patients with rectum adenocarcinoma (READ) (Supplementary file S1, Table S3). For these cancers, overall CD274 expression was much lower than in BRCA, LUAD and SKCM patients. Nevertheless, the generalized MLR model correctly predicted CD274 expression within a two-fold deviation from expectation for 94% of the CHOL patients and 98% of the READ patients (Fig. 2e,f). In conclusion, a generalized model developed by using combined data of common cancers demonstrated superior MLR extrapolation capacity and can thus be used to predict CD274 expression in other cancer types and to identify patients in whom expression is high relative to the observed TF activity.

### Mutations partially explain high CD274 expression

For patients in the underpredicted patient groups, the observed CD274 expression cannot be explained using only TFs as explanatory variables. This could be due to specific characteristics of the patients’ tumors, a primary candidate involving the genetic make-up of tumors. Therefore, we investigated whether mutations associated with increased CD274 levels could explain the underpredicted CD274 levels in those patients.

**Figure 3:**
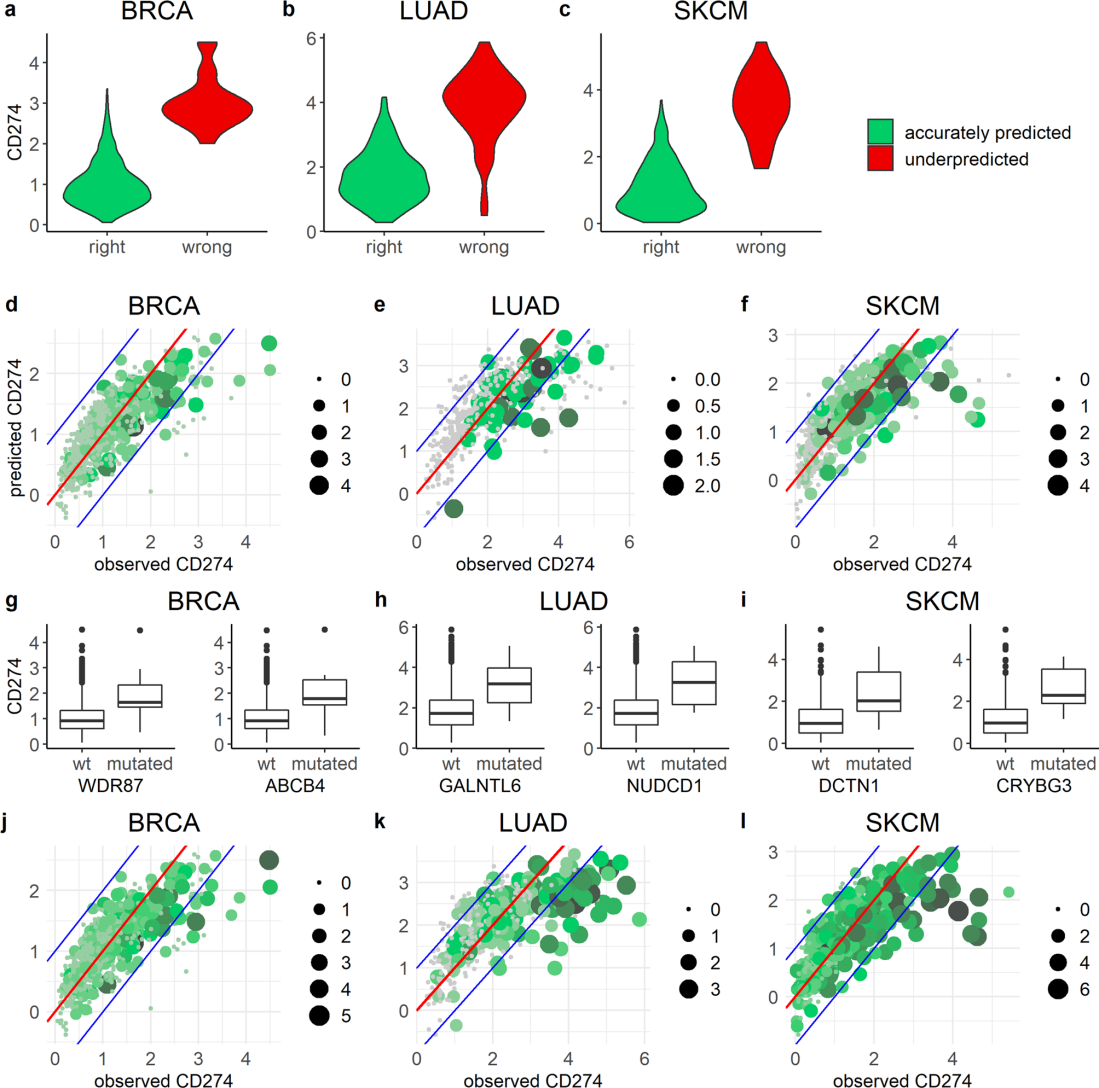
Mutations partially explain patients with high CD274 expression. (**a–c**) Violin plot comparing CD274 expression between accurately predicted and underpredicted patient groups. (**d–f**) Total mutation count in individual patients for earlier established critical genes that are based on only the accurately predicted group. (**g–i**) Examples of mutations leading to elevated CD274 expression based on the total patient population. (**j–l**) Total mutation count in individual patients for critical genes that are based on the total patient population. Both dot size and color indicates the log2 of the total number of mutations in critical genes. In all panels the cancer type is indicated above the panel.

On average, 77, 251, and 485 mutational events per patient were observed in BRCA, LUAD, and SKCM respectively. To identify which of these mutations could be relevant for increasing CD274 expression in the underpredicted group, we first tested which of the mutations were statistically associated with increased CD274, aiming to subsequently ask whether these mutations occur more frequently in the underpredicted than in the correctly predicted patient group. Since CD274 expression in the underpredicted group is on average higher compared to the accurately predicted group (Fig. 3a–c), we started by conducting the initial analysis to identify mutations associated with increased CD274 expression based on solely the accurately predicted group. We reasoned that residual variation in CD274 expression in this already well-predicted group, unexplained by TF activity, might be due to mutations, and that these specific mutations might occur more frequently in the underpredicted group. Mutations in 22, 9, and 33 genes were associated with significantly increased CD274 expression in BRCA, LUAD, and SKCM, respectively (*p* < 0.01, FC > 1.5) (Supplementary file 2, Sheet 3). Patients in the underpredicted group had an average of 0.6, 0.3, and 1.3 mutations in these critical genes in BRCA, LUAD, and SKCM, respectively, while the average in the accurately predicted group was 0.3, 0.1 and 0.9 mutations (Fig. 3d–f), i.e., only moderately different.

To investigate whether mutations identified based on the entire patient population could be more important determinants of CD274 expression than those based on just the accurately predicted group, we reiterated the analysis with the underpredicted patients included. Note that detecting potentially relevant mutations on the basis of solely the underpredicted group is precluded by the small patient number within this group. In this case, we found 36, 44, and 110 mutations in BRCA, LUAD, and SKCM, respectively (Supplementary file 2, Sheet 4 and Supplementary file S1, Fig. S4). In BRCA, mutations in WDR87 (*p* = 1.46e−5, FC = 1.82) and ABCB4 (*p* = 2.32e−4, FC = 1.98) showed the strongest relationship with elevated PD-L1 expression (Fig. 3g). In LUAD this was the case for GALNTL6 (*p* = 1.45e−4, FC = 1.63) and NUDCD1 (*p* = 5.41e−4, FC = 1.77) (Fig. 3h), and in SKCM for DCTN1 (*p* = 9.32e−5, FC = 2.04) and CRYBG3 (*p* = 4.47e−4, FC = 2.21) (Fig. 3i). Patients in the underpredicted group had an average of 2.6, 3.6, and 15.1 mutations in these critical genes, while this was only 0.5, 0.6, and 3.1 in the accurately predicted group (Fig. 3j–l), i.e., a substantial difference in abundance which was highly significant (BRCA: *p* = 2.2e−5, LUAD: *p* = 2.2e−16, and SKCM: *p* = 2.2e−9). In conclusion, mutations associated with increased PD-L1 levels occur more often in the underpredicted group, and we observed the strongest difference in abundance on the basis of selecting mutations for the entire patient group. This indicates that the mutations based on the underpredicted group are likely the most important ones with regard to elevated PD-L1 levels, and may have large effect.

### Low-abundant miRNAs partially explain high CD274 expression

**Figure 4:**
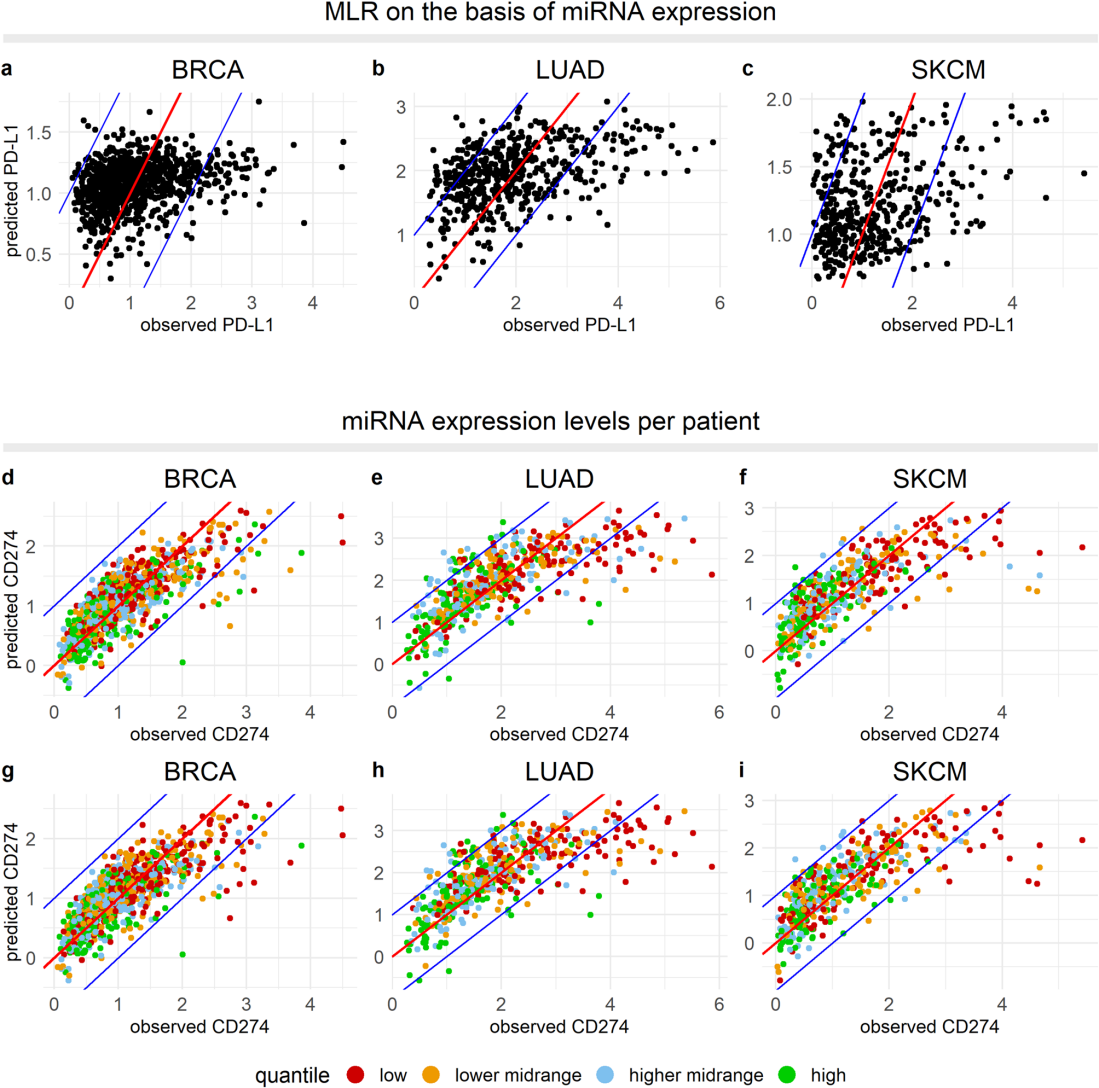
miRNAs partially explain patients with high CD274 expression. (**a–c**) MLR model based on the average expression of miRNAs associated with a negative regulation of CD274. (**d–i**) Patients were divided into quantiles based on the average expression of miRNAs identified as negative regulators of CD274 based on only the accurately predicted group (**d–f**), or based on the total patient population (**g–i**). Color indicates the quantile associated with a patient. In all panels the cancer type is indicated above the panel.

In addition to the role for mutations associated with increased CD274 expression, we investigated whether low levels of miRNAs known to target CD274, could provide an explanation for high CD274 levels in the underpredicted patients. To identify miRNAs associated with negative regulation of PD-L1 expression, we analyzed the expression levels of 1881 miRNAs. As before, we started this analysis based on only the accurately predicted group. Although the correlations were weak, we classified 24, 87, and 52 miRNAs as negative regulators of CD274 in BRCA, LUAD and SKCM, respectively (Supplementary file 2, Sheet 5). Approximately 29%, 15%, and 12% of the critical miRNAs in these cancer types also negatively regulated CD274 expression in at least one of the other cancer types. miRNAs from the let-7, mir-10, mir-1276, mir-17, mir-196, and mir-203 families were found to be negative regulators of CD274 in all three cancer types. As a test to see whether these miRNAs indeed play a role in the observed CD274 expression, we developed an MLR model including only the average expression of these miRNAs (Fig. 4a–c). The miRNAs clearly had predictive value for CD274 expression (BRCA, *p* = 2.2e−16; LUAD, *p* = 2.2e−16; SKCM, *p* = 1.6e−12), although the residual variation was much higher prediction was much worse than that based on TF activity (R^2^ values for MLR based on miRNA expression versus based on TF activity: BRCA, 0.09 vs. 0.60; LUAD, 0.20 vs. 0.51; SKCM, 0.11 vs. 0.57).

To visualize the miRNA expression amongst patients, the patient population was divided into quantiles based on the average expression of the critical miRNAs per cancer type (BRCA, low: < 3.68, lower midrange: = 3.68–3.89, upper midrange: 3.89–4.14, high: ≥ 4.14; LUAD, low: < 2.23, lower midrange: 2.23–2.40, upper midrange: 2.40–2.60, high: ≥ 2.60; SKCM, low: < 3.03, lower midrange: 3.03–3.23, upper midrange: 3.23–3.44, high: ≥ 3.44). In the underpredicted group, 19%, 56%, and 45% of the patients were classified in the lowest quantile, against 25%, 22%, and 24% in the accurately predicted group for BRCA, LUAD, and SKCM, respectively. Thus, a subset of critical miRNAs may contribute to high CD274 expression in LUAD and SKCM (Fig. 4d–f).

To investigate whether we could identify additional important miRNAs when basing the analysis on the whole patient population, we included the underpredicted patients in the first step and reiterated the analysis. In this case, we identified 29, 89, and 81 miRNAs as negative regulators of PD-L1 (Supplementary file 2, Sheet 6). We again divided the population into quantiles where we classified 48%, 63%, and 59% of the underpredicted patients in the lowest quantile, against 25%, 21%, and 23% of the accurately predicted group, a substantial difference (Fig. 4g–i; BRCA: *p* = 0.01, LUAD: *p* = 1.28e−6, SKCM: *p* = 0.0002). In conclusion, the expression of miRNAs associated with downregulation of PD-L1 was clearly lower in the underpredicted group, thus providing a potential explanation for a part of the patients within the underpredicted group. The finding that miRNAs that are selected on the basis of the accurately predicted group already show a clear effect suggests that the residual variability in CD274 expression within the accurately predicted group could also partially be due to these miRNAs.

### Mutations and low-abundant miRNAs jointly explain high CD274 expression

**Figure 5:**
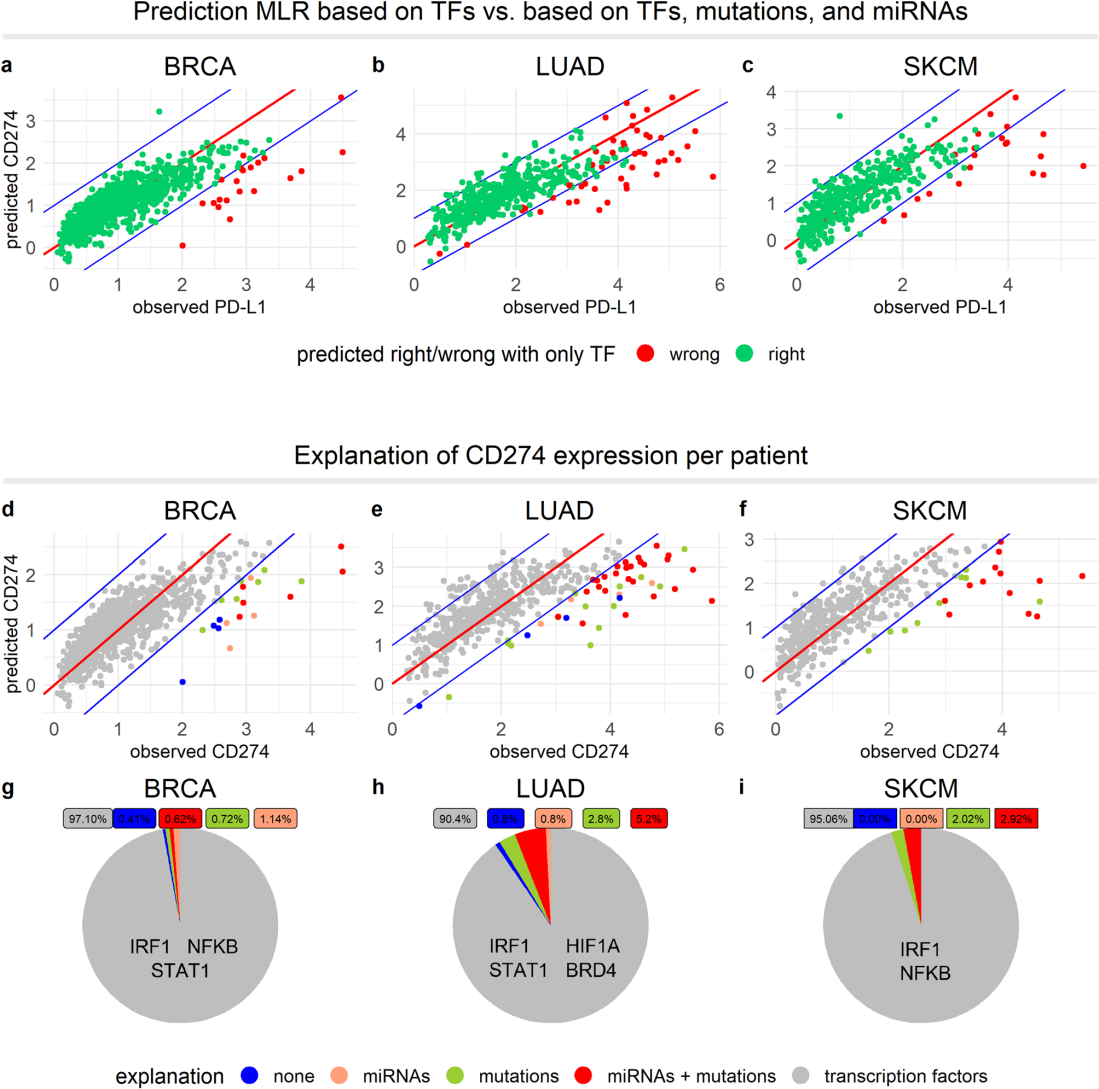
miRNAs, mutations and TF activity together explain CD274 expression in cancer patients. (**a**–**c**) MLR model based on TF activity, mutations, and miRNAs. Color indicates whether an individual patient was underpredicted in the MLR based on only TFs. (**d–f**) Results of MLR models with the patients colored according to how their CD274 expression can be explained. (**g–i**) Pie charts indicating the percentage of patients that can be explained by a specific factor. Significantly contributing TFs per cancer type are indicated within the grey regions. In all panels the cancer type is indicated above the panel.

Finally, to summarize which underpredicted patients could be explained by mutations and/or miRNAs, we developed an MLR model based on TF activity, the number of mutations, and the average miRNA expression. All factors were significant (*p* < 0.01), and improved the prediction of CD274, especially for LUAD (Fig. 5a–c). However, the predictive value of mutations and miRNAs within the MLR might not be optimal due to their possibly large effect, i.e., potential non-linearities not taken into account by an MLR approach. We therefore took the MLR predictions on the basis of TF activity as a basis and colored for individual underpredicted patients whether their CD274 expression could be explained by mutations and/or miRNAs (Fig. 5d–f). Mutations in a critical gene or low levels of critical miRNAs could be an explanation for the high CD274 levels in the majority of the patients in the underpredicted group. Specifically, in only 14% (BRCA), 8% (LUAD), and 0% (SKCM) of the patients in the underpredicted group, neither mutations nor miRNA can provide an explanation for the low CD274 expression, which is 0–1% of the total patient population in all three cancer types (Fig. 5g–i). Finally, we investigated whether the mutations and miRNAs identified from BRCA, LUAD and SKCM could provide an explanation for CHOL and READ patients (Fig. S5). Although the number of underpredicted patients with these cancers was too low for robust conclusions, indeed some of the mutations occurred within the identified genes. Overall, we conclude that CD274 expression can in the far majority of patients be predicted accurately by the activity of a small set of TFs. Moreover, the patients in whom the observed CD274 expression is clearly higher than expected based on TF activity, critical mutations and low expression levels of critical miRNAs can in most cases explain these high CD274 levels.

## Discussion

Prior research has indicated that multiple factors, ranging from TF activity within various pathways, mutations in critical genes, and low abundance of specific miRNAs influence PD-L1 expression^24^, but a quantitative picture was lacking. Therefore, we investigated to what extent TFs that are known to directly target the CD274 promotor can predict individual CD274 levels and whether there are dominant TFs. Our findings (summarized in Fig. 5g–i) illustrates that tumors have diverse pathways primarily involved in the regulation of PD-L1, and are consistent with prior findings^40^. Moreover, we found IRF1 to be a dominant inducer of CD274 expression in all three cancer types we studied, which is consistent with IFN-γ being a dominant cytokine driving PD-L1 expression in tumors^41^, because it primarily signals through the canonical type II interferon pathway, activating the TF IRF1^42–44^.

Amongst the differences in CD274-regulating TFs between cancer types, especially lung cancer stands out. Our finding that HIF1A induces CD274 expression in LUAD, but not in BRCA or SKCM, is in line with observed high HIF1A levels in both small cell and non-small cell lung cancers^37^. Additionally, cigarette smoking is linked to about 80–90% of lung cancer deaths, and proinvasive nicotine involves stabilization and activation of HIF1A^38,39^. Overexpression of HIF1A caused by smoking, could therefore potentially explain its dominant role in PD-L1 expression in LUAD patients, although the indisputable smoker/non-smoker group sizes were too small to confirm this (Supplementary file S1, Fig. S3). PD-1/PD-L1 inhibition therapies are more effective in smokers compared to non-smokers^45^, which has been linked to a higher tumor mutation burden (TMB) in smokers^46^. However, smoking-induced overexpression of HIF1A which leads to increased CD274 expression offers an alternative or additional explanation on why these therapies are more effective in smokers.

Besides being associated with high HIF1A expression, in lung cancer patients EGFR is a frequently mutated gene, causing increased levels of IκBα^47^. Consistently, PD-L1 expression is stronger in EGFR mutant NSCLC cells compared to wild-type EGFR NSCLC cells, and this is associated with increased expression levels of IκBα^48^. IκBα is a major IκB protein, which binds to NFKB dimers and sterically blocks the function of their nuclear localization sequence, causing their cytoplasmic retention and decreasing transcriptional activity of NFKB^49^. This could render NFKB a less important TF for PD-L1 expression in LUAD compared to BRCA or SKCM, as we found in our analysis. In contrast, BRD4 expression is frequently upregulated in non-small cell lung cancer (NSCLC) tissues^50^, which could lead to its increased importance in the regulation of CD274 in LUAD patients.

It is well established that IFN-γ signals through the JAK/STAT pathway, and targets the TFs IRF1 and STAT1. However, in melanoma cell lines IFN-y can also induce expression via the NF-kB pathway rather than via activity of STAT factors^51^. This potentially explains why STAT1 did not emerge from our analysis as a significant regulator of CD274 expression in SKCM. Although this discussion on dominance of TFs highlights that our approach correctly identified differential CD274 regulation between cancer types, our generalized model based on an ensemble of patients with different cancer types was also good at predicting CD274 expression. The excellent extrapolation of this generalized model to other cancer types for which fewer data are available provides confidence that the TFs IRF1, STAT1, NFKB, and BRD4 are in general dominant regulators of CD274 expression.

Importantly, the expression levels of the studied TFs gave a suitable prediction for CD274 expression in the far majority of patients, but not for a minority of ~ 2–10% of the patients. Genetic alterations within tumors may modulate the expression of immune markers, including PD-L1^52^. The small sample size of underpredicted patients precluded discovery of such mutations from this group alone, thus limiting our analysis. Nevertheless, based on the entire patient population we were able to identify several mutations that were statistically associated with elevated CD274 expression. For instance, LUAD tumors with high PD-L1 expression levels are frequently mutated in NUDCD1, which is involved in cell proliferation, migration, and invasion^53^. It exerts oncogenic functions via regulating the IGF1R-ERK pathway in NSCLC cells^54^. IGF1R activation mediates the activation of PI3K signaling, which regulates PD-L1 expression by either transcriptional or post-transcriptional mechanisms^55,56^. Activating mutations in the NUDCD1 gene could thus lead to an increased activation of this downstream pathway, causing an escalating expression of PD-L1. This example demonstrates that our identified mutations indeed may affect PD-L1 expression. Identification of these mutations provides groundwork for future studies to uncover functional implications of critical mutations in the regulation of PD-L1. Importantly, TCGA mutation data are restricted to coding regions, so we might have missed important mutations in non-coding DNA that are involved in the upregulation of CD274 expression. Future studies using whole genome sequencing data are thus needed to identify relevant mutations in non-coding regions.

Besides mutations in critical genes providing a potential explanation for high CD274 levels independent of TF activity, we also identified dozens of miRNAs as negative regulators of CD274 expression providing such an explanation. Out of the identified miRNAs, indeed some are known to negatively affect CD274 expression. For instance, the miRNA let-7 suppresses CD274 expression post-transcriptionally^57^. Furthermore, miR-203 binds the FGF2 protein, which normally promotes expression of PD-L1, leading indirectly to an inhibition of PD-L1^58,59^. Interestingly, we found that the miR-200 family, which is frequently mentioned as a direct regulator of PD-L1^60,61^, negatively regulated CD274 only in BRCA. Finally, consistent with a role of the PD-L1 3-UTR region to which miRNAs bind, structural variations in this region plays an important role in PD-L1 overexpression in tumor cells^62^.

In total, we found that mutations in critical genes, or low expression levels of critical miRNAs can provide an explanation for high CD274 levels in 80–100% of the patients in whom CD274 expression was not solely dependent on the expression levels of TFs. Factors that might mediate the high CD274 expression in the remaining unexplained patients include transcript stabilizers. Recently, it was found that EGFR signaling can rapidly stabilize PD-L1 mRNA via reduced activity of such stabilizers^30^. Thus, high EGFR levels or alternative means of increasing EGFR signaling might offer an explanation for the high CD274 levels in our underpredicted group. However, given that in the far majority of our studied patient group CD274 expression is already explained, we expect only a limited overall role of mRNA stabilizers for CD274 expression.

Knowing the underlying causes for high PD-L1 expression in a patient may in the future assist selection of patients suitable for PD-1/PD-L1 blockade. In this light, our underpredicted patient group is particularly interesting: the low TF activity relative to the CD274 expression in such patients could indicate absence of an ongoing (or suppressed) immune response which can be re-invigorated by therapy. These patients might therefore not be suitable for PD-1/PD-L1 monotherapy. However, our research was conducted only on mRNA level, which is not necessarily representative for the amount of PD-L1 on the membrane^63^. Thus, it is of high importance to extend our analysis in future to understand the dynamic relation between PD-L1 mRNA and protein. Approaches examining the dynamics of PD-L1 at both mRNA and protein level and of its regulators within single cells rather than using bulk data, will likely provide further insight into cell type specific effects^25^. Finally, further research is needed to identify the effect of treatment on our underpredicted patient group compared to accurately predicted patients. Such efforts may in the future contribute to the shaping of clear patient selection criteria for immune checkpoint therapy.

## Methods

### Analysis of The Cancer Genome Atlas (TCGA)

Data were retrieved and analyzed from the TCGA repository (http://cancergenome.nih.gov/) using *R* (version 4.0.2.). We employed the package *TCGAbiolinks* (version 2.16.4)^64^ to download and prepare level 3 RNA-seq and miRNA data for BRCA, LUAD, SKCM, CHOL, and READ patients. The human TCGA data that we reanalyzed were previously published as part of various studies, and in all cases this was done in accordance with relevant guidelines and regulations, including the obtainment of informed consent from all study participants and/or their legal guardians^65–71^.

The mRNA data represents expression levels in fragments per kilobase million (FPKM) normalized by gene length and total read count, and the miRNA data represents expression levels in reads per million (RPM), normalized by total read count. Each measurement in the resulting dataset was log2 transformed after adding the value 1 (to retain expression levels of zero). We used the package *miRBaseConverter* (version 1.12.0) to retrieve family information from the miRNA data. Mutational data was downloaded from cBioPortal and prepared by the package *maftools* (version 2.4.12)^72^, based on the *mutect2* somatic variant caller. We only included patients in our study for which both mutation spectrum and RNA-seq data were available, which led to a total of 970, 521, 445, 36, and 165 individual cases for BRCA, LUAD, SKCM, CHOL, and READ respectively. To characterize the patients used for our analysis, we also downloaded clinical data such as age, gender, therapy and outcome from the TCGA data portal using *TCGAbiolinks*^64^. For LUAD, CHOL, and READ approximately 90% of the patients were 50 years or older, with a median age of 66 years in all three groups. For BRCA and SKCM approximately 70% of the enrolled subjects were 50 years or older, with a median age of 58 in both groups. Most LUAD and CHOL patients were diagnosed in clinical stage I and most BRCA patients in clinical stage II, while this was more variable for SKCM, and READ patients. The average follow-up time was 39, 28, 59, 28, and 24 months in BRCA, LUAD, SKCM, CHOL, and READ, respectively, and 136, 188, 215, 18, and 27 patients died during this period (Supplementary file 1, Tables S2 and S3). Tumor samples were taken both before and during therapy and we do not distinguish between these options in our analysis. Moreover, we did not take into account the potential influence of factors such as age and sex on CD274 expression, because if such differences exist it seems likely that the influence would act through the investigated factors that we do already take into account (TF activity, mutations, miRNA expression). To test the difference in HIF1A expression and HIF1A TF activity between smokers and non-smokers in LUAD patients we compared the patients classified as current smokers, and life-time non-smokers using a two-sided t-test. Note that we did not consider patients falling in other categories because it was ambiguous what HIF1A levels should be expected for these subpopulations.

### Discriminant regulation expression analysis (DoRothEA) of target genes

The package *DoRothEA* (version 1.0.1)^73^ was used to find target genes of the TFs Myc, HIF1A/2A, c-Jun, IRF1, STAT1, STAT, and NFKB. Each TF regulates a set of genes, which is known as a regulon. DoRothEA has obtained these regulons from different types of evidence and they are accompanied by an empirical confidence level, ranging from A (most confident) to E (least confident)^74^. For our analysis, only target genes with the highest confidence were selected. Additionally, only the target genes which were unique amongst the considered TFs were used for further analysis (Supplementary file 2, Sheet 1). Note that target genes for BRD4 were not available in DoRothEA, hence we adopted genes put forward in a recent review^34^ (Supplementary file 2, Sheet 2)*.*

### T-distributed stochastic neighbor embedding (t-SNE)

To investigate patterns in the activity of TFs which are known to target the PD-L1 promotor, we used t-SNE, which constructs a low dimensional embedding of high-dimensional data^75^. The t-SNE plots were computed using the R package Rtsne using the expression levels of the independent target genes as a proxy for activity of TFs targeting the PD-L1 promotor, but excluding PD-L1 itself, as input data^76^. The perplexity was set to 30, and 3000 iterations were performed. We applied k-means clustering to the estimated TF activity to divide the patient population into four clusters, for which CD274 expression was subsequently visualized.

### Multiple linear regression analysis

To quantitatively describe the relationship between CD274 expression and the activity of its TFs, we employed multiple linear regression (MLR) models. The mean expression of the genes targeted by the TFs that potentially regulate PD-L1 expression were used as explanatory variables. The mean was calculated based on the normalized log2 transformed data (after addition of 1). The used MLR models have the general form:

1 $$\gamma_{i} = \beta_{0} + \beta_{1} x_{i1} + \beta_{2} x_{i2} + \cdots + \beta_{n} x_{in} + \varepsilon$$

Here, $\gamma_{i}$ corresponds to the dependent variable CD274. Each $x_{i}$ corresponds to the value of an explanatory variable (e.g., the average expression of IRF1 target genes), while $\beta i$ (with $i \in \left\{ {1,2, \ldots ,n} \right\}$) represents the corresponding regression coefficient, which is estimated by fitting the equation to the data using a least-squares approach. The intercept in the equation is described by $\beta_{0}$, and $\varepsilon$ is the model’s error term (residuals). We subsequently translated the regression coefficients to contributions (C) CD274 expression per TF (relative to that of other TFs) using the following formula:

2 $$C = \frac{{\beta_{1} x_{i1} }}{{\beta_{1} x_{i1} + \beta_{2} x_{i2} + \cdots + \beta_{n} x_{in} }}*100\%$$

This equation was applied per individual patient within a group, and from the distribution of relative contributions for all patients we determined the mean and standard deviation. Since the TFs that we included in our MLR, except for BRD4, are thought to increase PD-L1 expression, we used a bounded variable least squares (BVLS) solver to select TFs with a positive effect on CD274 expression. Thus, we set the lower bounds for all coefficients to zero, except for BRD4 and the intercept, and included only TFs with non-zero values, and a significance of *p* < 0.001 in our final model.

Patients in whom PD-L1 was predicted with a more than two-fold difference from the observed value were classified as part of the ‘underpredicted group’, i.e., for patients in this group the MLR model cannot predict CD274 expression using only TF activity. Similarly, patients in whom CD274 expression was predicted with a more than two-fold difference from the observed value form an ‘overpredicted group’. However, this group did not occur in BRCA, and was very small in LUAD and SKCM. Therefore, for the subsequent miRNA and mutation analysis we solely focus on the CD274 levels in the underpredicted group. Note that changing the threshold for the underpredicted group to a 1.5-fold (instead of two-fold) difference between predicted and observed values led to slightly altered lists of critical mutations and miRNAs, yet this did not change the conclusions with respect to presence of these mutations and miRNAs in the underpredicted group relative to their presence in the well-predicted group (not shown).

### Collinearity diagnostic test

Multicollinearity refers to the situation where two or more independent variables in a regression model are correlated, which may cause unreliability of the model parameter estimates and should therefore be avoided^77^. Multicollinearity in our final regression model was diagnosed using the Variance Inflation Factors (VIF) diagnostic test. In particular, the VIF for the predictor is:

3 $$VIF_{i} = 1/\left( {1 - R_{i}^{2} } \right)$$

where $R_{i}$ represents the R^2^-value obtained by regressing the $i{\text{th}}$ independent variable on the remaining predictors. When VIF is equal to 1, the $i{\text{th}}$ independent variable is not correlated to the other predictors, which implies that there is no multicollinearity in the regression model. Generally, a VIF above 4 indicates that coefficient estimation is problematic, whereas for a VIF higher than 10 there is substantial multicollinearity which needs to be corrected^78,79^.

### Model development for extrapolation

To create a general linear regression model which can be used for extrapolation to other cancer types, the BRCA, LUAD, and SKCM datasets were combined, and subsequently divided into a training set (80%) and a test set (20%) by randomly selecting patients independent of the cancer type of origin. The estimated coefficients associated with all TFs having a non-zero regression coefficient and a significant effect (*p* < 0.001) on CD274 expression were included in the final model as based on the training set. We subsequently used these coefficients both for evaluation on the test set and for extrapolation to other cancer types (CHOL and READ)*.*

### Statistical tests

To explain the high CD274 expression in the patient group with underpredicted CD274 expression, we identified mutations associated with elevated CD274 levels based on the TCGA data. These mutations were selected by comparing CD274 expression per mutational status (wild-type or mutated) of each gene. We first performed this analysis for the patients with accurately predicted CD274 expression (based on TF activity), and subsequently reiterated this for the total patient population. We did not distinguish mutations based on their exact location or type, and cases where multiple mutations occurred in the same gene within a single patient were considered as one mutational event. We only considered mutations with a prevalence of over 1% and leading to a CD274 fold change (FC) of > 1.5 and *p* < 0.01 (based on a two-sided nonparametric Mann–Whitney U test). Subsequently, we studied whether the prevalence of these selected mutations was different between the underpredicted group and the accurately predicted group using a Mann–Whitney U test.

To consider the potential role of post-transcriptional regulation of CD274 by miRNAs, we identified miRNAs associated with CD274 downregulation in a similar manner as for the approach based on mutations described above. The correlation between individual miRNAs and PD-L1 expression was evaluated using a two-sided Pearson product-moment correlation test. As for the mutations, this analysis was first performed for the accurately predicted subgroup only (based on TF activity), and subsequently reiterated for all patients. All miRNAs with negative coefficients and a statistical significance of *p* < 0.01 were accepted as down-regulators of CD274. Subsequently we conducted a Mann–Whitney U test to compare the expression of miRNAs between the underpredicted group and the accurately predicted group.

## Supplementary Information

Supplementary Information 1.

Supplementary Information 2.

Supplementary Legends.

## Data Availability

The datasets analyzed during the current study are available in The Cancer Genome Atlas repository, http://cancergenome.nih.gov/.

## Abbreviations

ICI: Immune checkpoint inhibitors
PD-1: Programmed cell death protein 1
PD-L1: Programmed cell death protein ligand 1
TF: Transcription factor
miRNA: MicroRNA
BRCA: Breast invasive adenocarcinoma
LUAD: Lung adenocarcinoma
SKCM: Skin cutaneous melanoma
TCGA: The Cancer Genome Atlas
MAbs: Monoclonal antibodies
MLR: Multiple linear regression
t-SNE: T-distributed stochastic neighbor embedding
