# Supplementary material for: Quantifying the contribution of transcription factor activity, mutations and microRNAs to CD274 expression in cancer patients
Source: Sci Rep. 2022 Mar 14;12:4374. doi: 10.1038/s41598-022-08356-0 (PMC8921511; doi:10.1038/s41598-022-08356-0)
Supplement: Supplementary file 1 — Supplementary Information 1. [file 41598_2022_8356_MOESM1_ESM.pdf]

Supplemental figures

Quantifying the contribution of transcription factor activity, mutations and microRNAs to CD274 expression in cancer patients

Imke B. Bruns[1], Joost B. Beltman[1]*

[1] Division of Drug Discovery and Safety, Leiden Academic Centre for Drug Research, Leiden University, Leiden, Netherlands

*** Correspondence:**

Joost B. Beltman

j.b.beltman@lacdr.leidenuniv.nl

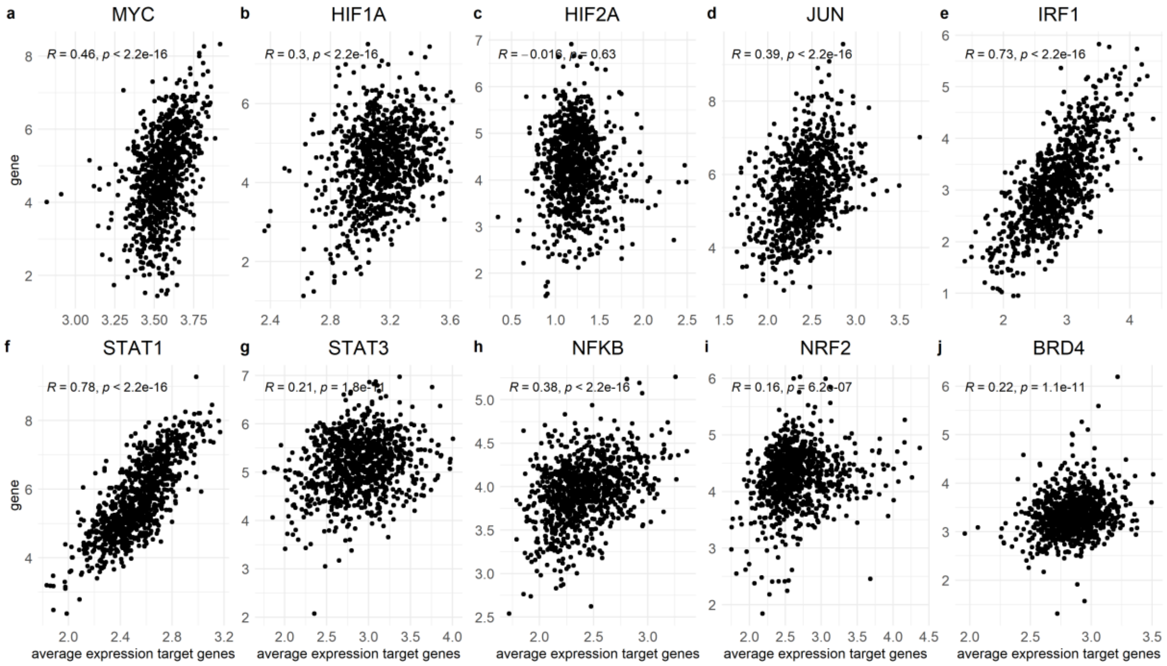

Supplemental figure 1: Expression of TFs versus their target genes in BRCA patients. (a-j) The studied TFs are indicated above the panels, as are the Pearson correlation coefficient (*R*) and *p*-values.

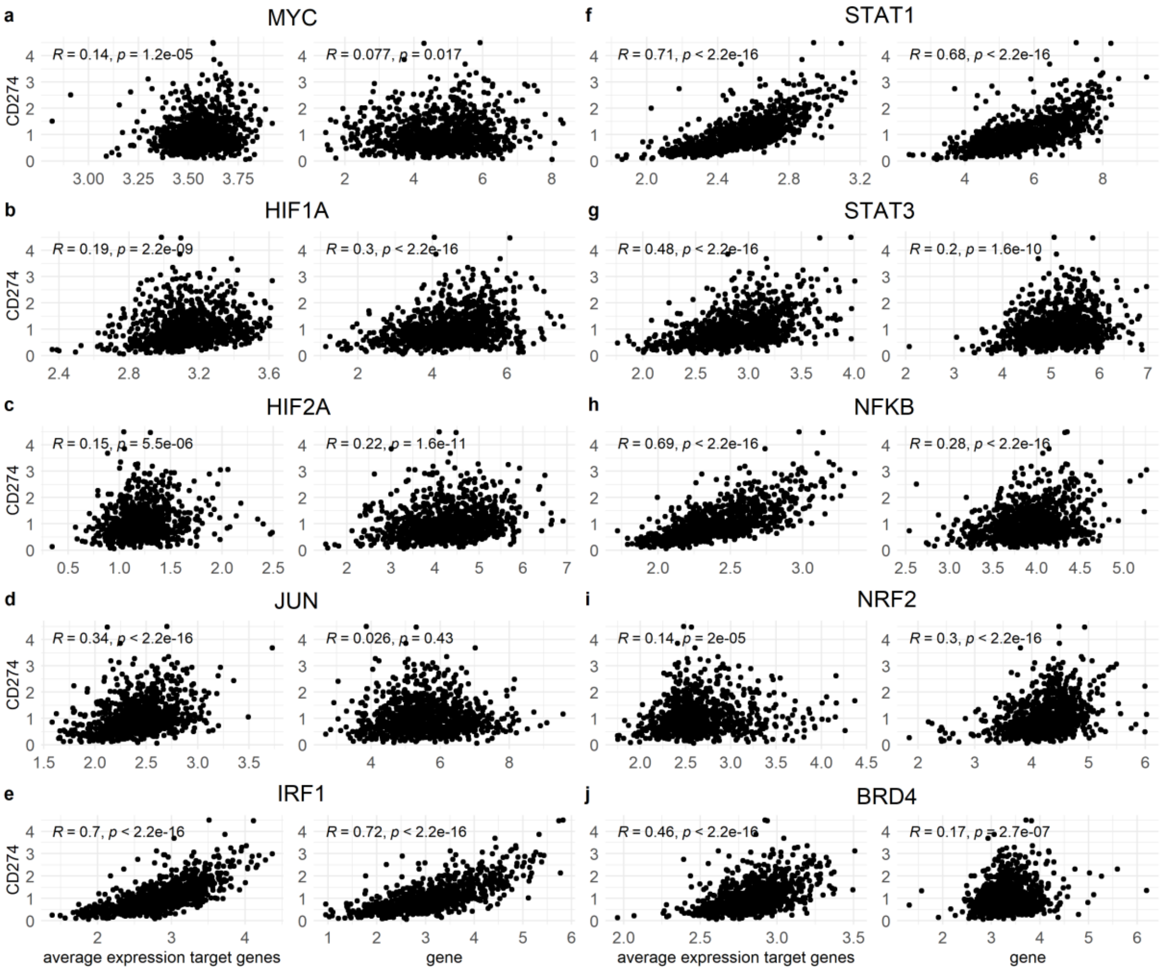

Supplemental figure 2: CD274 expression as a function of estimated TF activity and of TF abundance. (a-j) Shown are plots for the observed expression of CD274 versus the activity of indicated TFs (left panels) and versus the directly observed expression of those factors (right panels). Pearson correlation coefficient (*R*) and *p*-value are indicated above panels.

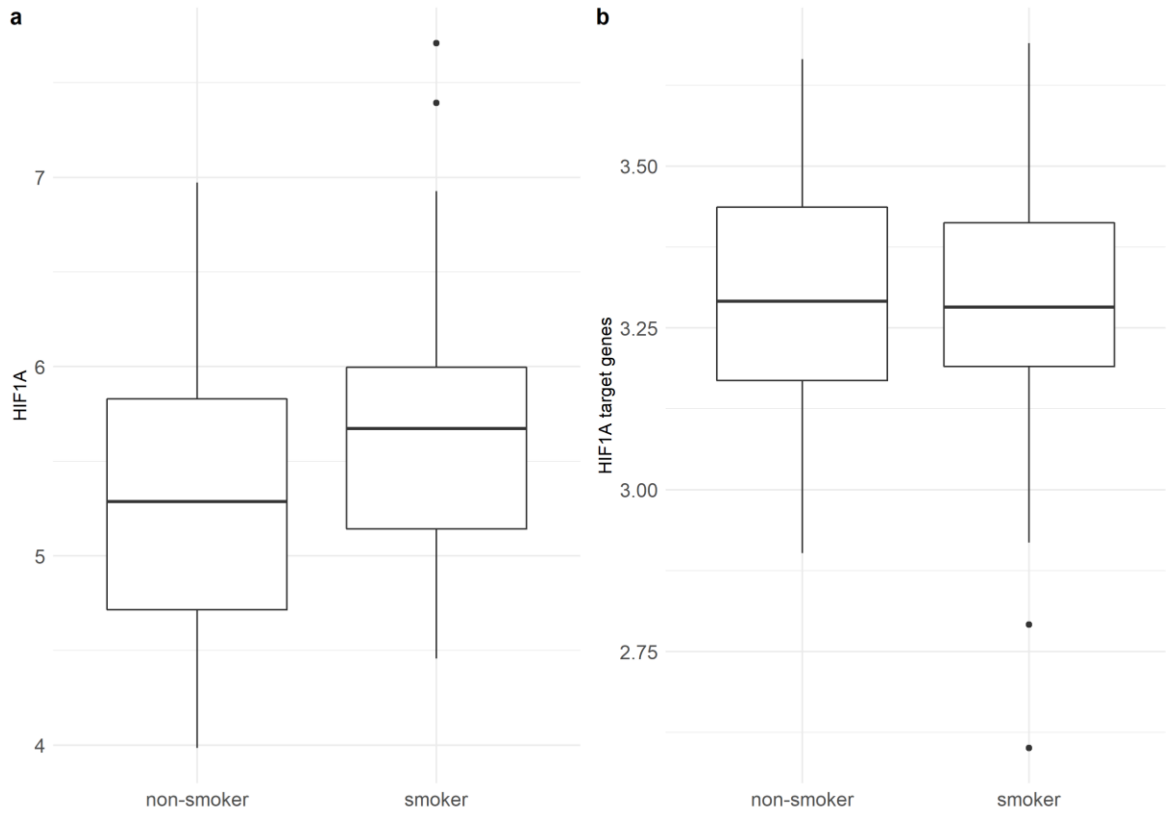

Supplemental figure 3: Expression of HIF1A (a) and HIF1A target genes (b) in smoking and non-smoking LUAD patients. LUAD patients were divided based on smoking habits, resulting in 21 non-smokers and 46 smokers. Note that HIF1A expression and HIF1A TF activity as based on target gene expression is not significantly different between smokers and non-smokers, although there is a tendency for such a difference in HIF1A expression.

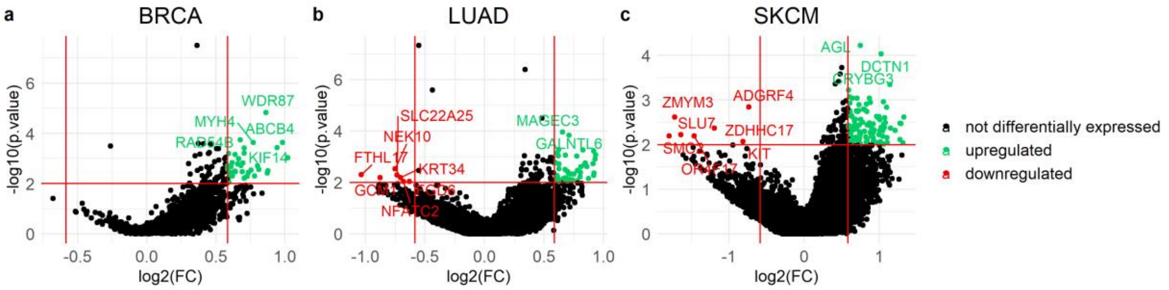

Supplemental figure 4: Mutations significantly increasing CD274 expression. Mutations in 36, 44, and 110 genes were associated with significantly increased CD274 expression ($p < 0.01$, FC > 1.5) in BRCA (a), LUAD (b), and SKCM (c), respectively.

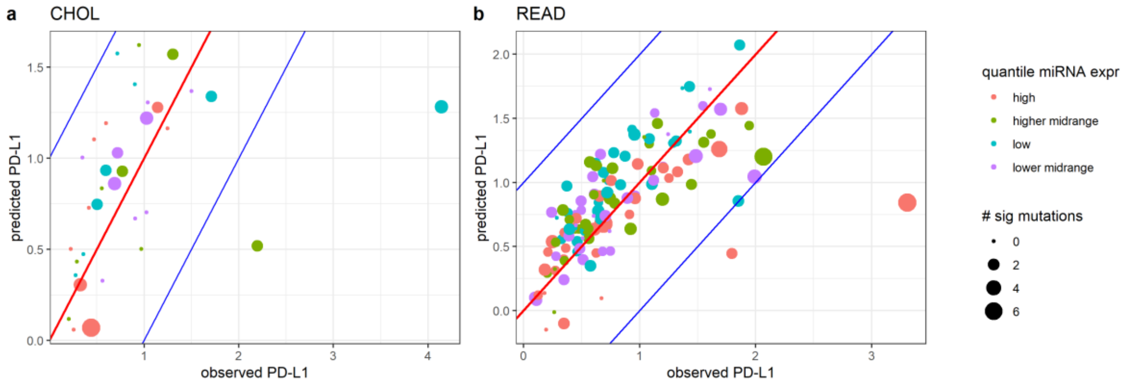

Supplemental figure 5: generalized multiple linear regression model applied to CHOL (a) and READ (b) patients. For individual patients with mutation and miRNA data available in TCGA, we indicated the number of mutations and miRNA expression by symbol size and colour, respectively.

Supplemental tables

	BRCA	LUAD	SKCM	Generalized
**MYC**	-	-	-	-
**HIF1A**	-	1.2112	-	-
**HIF2A**	-	-	-	-
**JUN**	-	-	-	-
**IRF1**	3.2944	2.8746	1.8513	3.2060
**STAT1**	2.8205	2.5226	-	3.5758
**STAT3**	-	-	-	-
**NFKB**	2.3192	-	1.8513	2.5208
**NRF2**	-	-	-	-
**BRD4**	-	1.4170	-	2.3760

Supplemental table 1: Variance Inflation Factors (VIF) diagnostic test data. Note that all VIF values are lower than 4, which indicates that the selected variables of the model are not highly correlated. A missing VIF (i.e., a dash) indicates that the given transcription factor was not included in the final model.

	Characteristics	BRCA Participants (n=970)	(%)	LUAD Participants (n=521)	(%)	SKCM Participants (n=445)	(%)
**Sex**	Male	959	99	243	47	275	62
	Female	11	1	278	53	170	38
**Age**	< 25	0	0	0	0	10	2
	≥ 25 < 50	274	28	33	6	119	27
	≥ 50 < 75	576	60	378	73	238	54
	≥ 75	120	12	91	17	72	16
	N/A			19	4	6	1
**Clinical stage**	I	163	17	289	55	73	16
	II	565	58	119	23	128	29
	III	210	22	83	16	163	37
	IV	19	2	24	5	24	5
	N/A	13	1	6	1	57	13
**Therapy**	Ancillary	18	2	3	1	3	1
	Chemotherapy	505	52	166	32	69	15
	Immunotherapy	22	2	5	1	70	16
	Targeted molecular therapy	25	3	10	2	12	3
	Hormone therapy	446	46	0	0	5	1
	Other	20	2	4	1	22	5
	N/A	5	1	333	63	263	59
**Response**	pCR	191	20	54	10	33	7
**Death event**		136	14	188	36	215	48

Supplemental Table 2: Clinical characterization of BRCA, LUAD and SKCM patients from the TCGA database.

	Characteristics	CHOL Participants (n=36)	CHOL (%)	READ Participants (n=165)	READ (%)
**Sex**	Male	16	44	90	55
	Female	20	56	75	45
**Age**	< 25	0	0	0	0
	≥ 25 < 50	4	11	20	12
	≥ 50 < 75	27	75	114	69
	≥ 75	5	14	31	19
	N/A				
**Clinical stage**	I	19	53	30	18
	II	9	25	51	31
	III	1	3	51	31
	IV	7	19	24	15
	N/A			9	5
**Therapy**	Ancillary	0	0	10	6
	Chemotherapy	9	25	77	47
	Immunotherapy	0	0	1	1
	Targeted molecular therapy	0	0	9	5
	Other				
		2	6	14	8
	N/A				
		25	70	54	33
**Response**	pCR	2	6	59	36
**Death event**		18	50	27	16

Supplemental Table 3: clinical characterization of patients from TCGA dataset (CHOL, READ).
