# Supplementary material for: Quantifying the contribution of transcription factor activity, mutations and microRNAs to CD274 expression in cancer patients
Source: Sci Rep. 2022 Mar 14;12:4374. doi: 10.1038/s41598-022-08356-0 (PMC8921511; doi:10.1038/s41598-022-08356-0)
Supplement: Supplementary file 3 — Supplementary Legends. [file 41598_2022_8356_MOESM3_ESM.docx]

**Supplementary file 1:** Supplementary material. This file includes Supplementary Figure 1-4 and Supplementary Table 1-3.
**Supplementary file 2:** Data sheets. This file contains data sheets with the following content: target genes identified by DoRothEA, BRD4 target genes, mutations associated with increased CD274 levels based on the right predicted group, mutations associated with increased CD274 levels based on the whole patient population, miRNAs identified as negative regulators of CD274 based on the right predicted group, miRNAs identified as negative regulators of CD274 based on the whole patient population.
